# Next of kin's experiences of healthcare encounters in the context of severe self-harm: “We know them best”

**DOI:** 10.1080/17482631.2026.2712680

**Published:** 2026-08-12

**Authors:** Kenneth Haugjord, John-Kåre Vederhus, Ottar Ness, Kristine Dahl

**Affiliations:** a District Psychiatric Center, Østre Agder, Sørlandet Hospital, Kristiansand, Norway; b Department of Psychosocial Health, University of Agder, Grimstad, Norway; c Addiction Unit, Sørlandet Hospital, Kristiansand, Norway; d Norwegian University of Science and Technology (NTNU), Trondheim, Norway

**Keywords:** Next of kin, self-harm, epistemic injustice, recognition, mattering, involvement

## Abstract

**Purpose:**

Policies in Norway and similar countries emphasize the inclusion of next of kin in healthcare. However, research shows that many experience limited involvement and recognition. This study explores how next of kin to individuals with severe self-harm experience and make sense of healthcare encounters in a Norwegian context.

**Methods:**

The study draws on in-depth interviews with 15 parents, partners, and siblings of individuals engaging in severe self-harm. Reflexive thematic analysis was employed, guided by a phenomenological sensibility.

**Results:**

Three interconnected themes illuminated different aspects of participants’ encounters with healthcare services. “In the hands of the system” captures experiences of navigating the healthcare system while concerned for a loved one’s wellbeing. “Who do I become when excluded” highlights participants' experiences of their experiential knowledge being marginalized and how emotional involvement appeared to undermine their credibility. “The ones who stand by” reflects concerns about their own wellbeing and that of the wider family, alongside a desire for greater recognition and support.

**Conclusions:**

The results suggest that healthcare encounters involve not only support and participation but also recognition of next of kin’s experiential knowledge and needs, which appears to shape their sense of legitimacy and ability to remain involved in care.

## Introduction

Self-harm can be understood as intentionally inflicting injury on oneself, regardless of suicidal intent (Hawton et al., 2012; Moran et al., 2024). It represents a significant global public health concern, particularly among adolescents (Moran et al., 2012; Tan et al., 2025), and is often associated with psychosocial difficulties, mental illness, and histories of trauma (Mcevoy et al., 2023; Moran et al., 2024). Self-harm may also involve substantial societal and healthcare-related costs (Langjord et al., 2023; Moran et al., 2024).

In situations where self-harm becomes persistent or severe, close family members and next of kin are often deeply affected and involved as they navigate ongoing concern and responsibility (Curtis et al., 2018; Martin et al., 2024). A substantial body of research has demonstrated that living alongside someone who self-harms may involve considerable emotional, relational, and practical strain for next of kin (Ferrey et al., 2016; Martin et al., 2024; Simes et al., 2022; Zhao et al., 2023). Next of kin frequently describe anxiety, guilt, shame, exhaustion, and ongoing worry, alongside disruptions to sleep, work, education and social relationships (Curtis et al., 2018; Martin et al., 2024; Mughal et al., 2022; Simes et al., 2022; Simon et al., 2025; Spillane et al., 2020; Townsend et al., 2021). Research suggests that self-harm may affect family relationships and dynamics, including marital strain and disruptions within family life, while siblings may experience reduced parental attention alongside their own emotional reactions to the situation (Curtis et al., 2018; Ferrey et al., 2016; Townsend et al., 2021; Zhao et al., 2023).

Alongside these documented burdens and strains, research has consistently pointed to the need for more targeted and sustained support for next of kin in the context of self-harm (Krysinska et al., 2020; Martin et al., 2024; Mughal et al., 2022; Zhao et al., 2023). Studies have highlighted a range of unmet needs, including information about self-harm, treatment pathways, and how best to support their loved one (French et al., 2024; Fu et al., 2020; Krysinska et al., 2020; Mughal et al., 2022; Townsend et al., 2023). Previous research has also emphasised the need to support next of kin themselves in managing emotional distress, uncertainty, guilt, and anxiety, as well as in balancing caregiving responsibilities with their own wellbeing and self-care (Cação-Dias et al., 2026; Curtis et al., 2018; Martin et al., 2024; Townsend et al., 2021). In addition, several authors have called for more family-oriented approaches that acknowledge the broader relational and family challenges associated with living alongside self-harm (Ferrey et al., 2016; Kelada et al., 2016; Simes et al., 2022; Townsend et al., 2021). Relatedly, recent qualitative studies of voluntary brief admission for individuals who self-harm suggest that this type of intervention may also positively influence the experiences of next of kin (Hultsjö et al., 2023; Lindkvist et al., 2024; Värnå et al., 2025).

While much of the research literature has focused on how healthcare services may better support next of kin, policy documents and clinical guidelines increasingly describe next of kin not only as recipients of support but also as important contributors to care (Aass et al., 2025; National Institute for Health & Care Excellence, 2022; Norwegian Directorate of Health, 2024). Encounters with healthcare services may therefore offer opportunities not only for support and guidance, but also for involvement, collaboration, and the sharing of knowledge and perspectives. (Mughal et al., 2022; Stewart et al., 2018; Townsend et al., 2023; Zhao et al., 2023).

However, studies consistently report that many next of kin experience mental healthcare services as falling short of these ambitions. Participants often describe feeling excluded, unheard, and insufficiently involved in care (Abou Seif et al., 2022; Cação-Dias et al., 2026; Dietrich et al., 2025; Lindgren et al., 2010; Møllerhøj, 2022; Stewart et al., 2018; Zhao et al., 2023). Research further suggests that these experiences may concern not only access to support and participation, but also whether next of kin's knowledge, perspectives, and contributions are acknowledged and taken seriously within care processes and clinical decision-making (Cação-Dias et al., 2026; Dietrich et al., 2025; Stewart et al., 2018; Townsend et al., 2023).

Through close and ongoing involvement in everyday life, next of kin may develop detailed knowledge of their loved one's history, everyday functioning, patterns of distress, and what may help or worsen their wellbeing (Hughes et al., 2017; Lindgren et al., 2010; Stewart et al., 2018). Understanding how next of kin experience bringing such knowledge and perspectives into healthcare encounters may therefore provide insight into how their contributions and involvement are understood and accommodated within care.

Although previous research has documented unmet support and information needs, as well as experiences of exclusion among next of kin, less attention has been directed toward how next of kin themselves experience and make sense of contributing their knowledge, perspectives, and experiences within healthcare encounters. Qualitative research attentive to participants' interpretations and meaning-making may deepen understanding of these encounters and inform how healthcare services engage with, recognise, and support next of kin. The guiding research question of the present study was: *How do next of kin experience and make sense of encounters with healthcare services in the context of severe self-harm?*

## Methods

### Design

This qualitative study was situated within an interpretivist paradigm (Cuthbertson et al., 2020) and was informed by a phenomenological orientation attentive to participants' lived experiences and meaning-making (Dahlberg et al., 2007; Van Manen, 2014). Within this perspective, experience is understood as something people describe and give meaning to in their everyday contexts.

Reflexive thematic analysis (Braun & Clarke, 2019, 2022a, 2022b) guided the analytic process. The study employed a theory-informing inductive approach (Varpio et al., 2020). Within reflexive thematic analysis, themes are understood as interpretive patterns developed through the researcher’s active engagement with participants’ accounts, and researcher subjectivity is treated as a resource in knowledge production. Accordingly, we understand our results as developed through engagement with participants and their stories rather than as findings that simply reside in the data (Braun & Clarke, 2019; Finlay, 2005). This design allowed us to attend to how participants described and interpreted their encounters with the healthcare system while developing situated interpretations across accounts.

### Participants and recruitment

The participants were 15 next of kin, all of whom were family-members, closely involved with a child, partner, or sibling who had struggled with severe self-harm for between 5 and 15 years. Participant and contextual characteristics are summarised in Table 1.

**Table 1.** Participant and contextual characteristics.

Participants	
Number of participants	15 next of kin
Relationships represented	7 mothers, 2 fathers (including 1 stepfather), 3 partners, 3 siblings
Participant age range	Early 20s to mid-60s
**Context of the loved one's situation**	
Typical onset	Late childhood or early adolescence
Duration	Approximately 5–15 years
Nature of self-harm	Included life-threatening injuries, suicide attempts, and lasting disabilities
Healthcare context	Ongoing contact with outpatient and inpatient mental health services
Age at recruitment	All individuals who self-harmed were adults (18 years or older)

Participants were purposively recruited to capture information-rich perspectives (Malterud et al., 2016) relevant to the study’s focused aim of exploring next of kin’s experiences during healthcare encounters in the context of severe self-harm. The first author contacted local services working with individuals who self-harm in and outside hospital settings and informed them about the study. Only next of kin of adults (18 years or older) who self-harmed were eligible for participation. In most cases, healthcare professionals informed individuals struggling with self-harm about the study. Next of kin were approached only after the person who self-harmed and their next of kin had agreed that the next of kin could be approached about the study. In some cases, the person who self-harmed informed their next of kin about the study, and interested next of kin contacted the first author directly. In other cases, interested next of kin were contacted by telephone by the first author after agreeing to be contacted.

Five additional participants were recruited through previously interviewed participants when other next of kin in the same families were identified as relevant to the study.

Inclusion was based on a pragmatic and clinically informed understanding of severe self-harm among the participants’ loved ones, drawing on duration, form, and patterns of healthcare contact. Following Malterud et al. (2016) concept of information power, the final sample size was considered adequate given the study’s focused aim, the specificity of the sample, and the depth and richness of the interview material.

### Data collection

The project group developed a semi-structured interview guide containing broad themes that supported an open dialogue about participants’ experiences as next of kin. The interviews explored participants’ everyday lives, family dynamics, understandings of their loved one’s difficulties, experiences of sharing their situation, and encounters with healthcare services. While the interviews explored several aspects of participants' experiences as next of kin, the analyses reported here focus on encounters with healthcare services.

In-depth interviews were conducted by the first author (KH), with two co-authors (JKV and KD) attending one interview each to facilitate a shared familiarity with the material within the research team.

Twelve participants were interviewed twice, while follow-up interviews were not conducted with three participants due to practical circumstances. This follow-up- approach enabled deeper exploration of topics the interviewer or participants wished to elaborate on, including reflections that emerged between interviews. There was no predefined interval between interviews, although time was allocated for transcription and preliminary analysis. Most follow-ups were conducted within a few months of the initial interview. One parent couple was interviewed together in both the initial and follow-up interviews.

Most interviews were conducted in hospital offices in accordance with participant preference. One interview was conducted in an office facility provided by the participant. Two participants were interviewed via video due to geographic distance. Video interviews were conducted using a secure digital platform provided by the hospital where the study was conducted. Initial interviews typically lasted around 90 minutes. Follow-up interviews varied in length but were generally shorter than the initial interviews.

All interviews were audio-recorded and transcribed verbatim by the first author with assistance from open-source speech recognition tool Whisper (Whisper, n.d.) used within the University of Oslo’s secure TSD platform (Services for Sensitive Data). Anonymized transcripts were uploaded to NVivo (version 14), for data management. Anonymized excerpts were later translated into English for publication.

### Data analysis

The analytical process was iterative and reflexive, involving repeated cycles of reading, coding, and theme development (Braun & Clarke, 2022a). Consistent with the phenomenological orientation of the study, attention was directed toward participants' descriptions of lived experience and meaning-making. Following the six phases of reflexive thematic analysis described by Braun and Clarke (2022a), the first author repeatedly read all transcripts to become familiar with the material and wrote brief notes to stay actively engaged with the data. Two co-authors read selected transcripts to gain an overview of the material. Initial coding by the first author was predominantly semantic in order to stay close to participants’ accounts. Codes that appeared to overlap or capture similar aspects of participants’ experiences were then grouped into preliminary themes. Themes were treated as analytic constructions developed through the researchers’ engagement with the data (Braun & Clarke, 2022a). This process initially generated eight potential themes. Through dialogue with co-authors and user representatives, the themes were reviewed and refined, with attention to overlap and how they could be combined or reorganised to better capture central aspects of participants’ experiences. This process resulted in three final themes. Consistent with the theory-informing inductive approach (Varpio et al., 2020), theoretical perspectives were gradually engaged to deepen and nuance the interpretation of the material. Writing the report intersected with the final stages of the analysis. One author (ON) was particularly involved in the development of the discussion.

### Reflexivity and user representative involvement

Working within an interpretive approach, we understand that knowledge is shaped by researchers’ perspectives, experiences, and values (Finlay, 2002). The research team includes a male PhD student and psychologist with a specialisation in community psychology, a female clinical psychologist with expertise in qualitative research, a registered male nurse and professor with a background in addiction medicine, and a male family therapist and professor in counselling. Together, these professional backgrounds informed our sensitivity to the clinical, relational, and experiential dimensions of the material. A shared interest in involvement and co-creation in social and clinical work also shaped the theoretical perspectives engaged during the study.

In addition to our professional backgrounds, we reflected on how personal experiences, including being next of kin, shaped our approached the material. For example, before engaging in the formal analysis, the research team held a dedicated discussion to identify and reflect on the professional and personal experiences we brought to the study and how these might shape our attentiveness to particular meanings in the data. Rather than treating these experiences as bias, they were acknowledged and discussed within the research team as part of ongoing reflexive work, and informed attentiveness to particular meanings while requiring critical reflection. This was particularly important when participants' accounts evoked strong emotional and embodied responses within the research team. These responses were discussed explicitly throughout the analytic process as opportunities to consider how they might both illuminate and constrain our understanding. User involvement also formed part of this reflexive orientation and was informed by principles recognising lived experience as a valuable source of knowledge, as well as participatory traditions emphasising dialogical and collaborative knowledge production (Borg & Askheim, 2010; Greenhalgh et al., 2016).

Two individuals were engaged throughout the study: one with lived experience of self-harm and one with lived experience as next of kin to someone with severe self-harm. They contributed to the early planning of the study and development of the interview guide. The first author met with these individuals regularly to discuss ongoing analytic work, and they were invited to reflect on developing interpretations. They were involved in a consultative and dialogical capacity, and did not read transcripts or participate in coding. Their reflections offered nuanced or expanded interpretations by highlighting underemphasised dimensions and variation in next of kin experiences. User involvement was not intended as validation, but to support reflexive engagement and strengthen the experiential grounding of the analysis. Their contributions are acknowledged in the Acknowledgements.

### Ethics

The study was approved by a regional ethics committee (REK, reference number 541067). Participation was voluntary. Next of kin received written and oral information about the study and provided written informed consent prior to participation. They were informed about the study's purpose, the first author's role as a PhD candidate and employment at the local hospital, what participation involved, their right to withdraw at any time without consequences, the confidential handling of their data, and available support services if participation evoked distress. Participants were assured of confidentiality. Several participants noted limited opportunities to speak about their experiences and expressed appreciation. Some described the conversations as emotionally demanding, but none reported regretting their participation or perceiving the emotionality as predominantly negative.

Several individuals with a history of self-harm who were approached declined to give consent for their next of kin to participate. We view this as supporting the ethical decision to require consent, as such relationships may be relationally fragile and complex.

## Results

The analysis developed three themes that illuminated different, though interconnected, aspects of next of kin’s encounters with healthcare, including their efforts to contribute, be involved, and have their own experiences acknowledged. Inspired by a participant's reflection that they knew their loved one best, the title “we know them best,” conveys the perspective from which participants described their encounters with healthcare, shaped by their lived experiences of supporting a loved one who self-harmed and the everyday realities this created for themselves, their loved one, and the wider family. The first theme, “In the hands of the system,” concerns how participants navigated entrusting their loved one to care while adapting to institutional structures and expectations under conditions of vulnerability. The second theme, “Who do I become when excluded,” focuses on experiences of limited recognition of next of kin’s experiential knowledge and involvement and how these experiences shaped their sense of self, credibility, and role. The third theme, “The ones who stand by,” shifts attention to participants’ own needs and those of the wider family, highlighting how next of kin often felt overlooked as persons in their own right.

### Theme 1: in the hands of the system

This theme concerns how participants described placing their loved one in the care of healthcare services while they themselves were subject to the system’s structures, expectations, and constraints. Entering healthcare meant adapting to an unfamiliar and often powerful system while entrusting it with the care of someone deeply important to the participant. This process was marked by vulnerability and uncertainty alongside a persistent sense of responsibility that was difficult to set aside. As healthcare professionals assumed responsibility for treatment and care, participants described how their concern for their loved one and sense of obligation to safeguard them remained. Many participants described staying closely involved throughout different stages of care, especially when their loved one was unable to express their needs. This involvement often took the form of advocating, monitoring, and pushing for action when they felt something was at stake. Illustrating this dynamic, one father described how he experienced a strong need to act on behalf of his daughter:

We’ve tried to push as much as we could within the system to make progress with her treatment, pushing for action to ensure things didn’t stall. If our daughter had been the one to set the pace, there would have been no progress because she didn’t express anything. She didn’t articulate any needs, such as saying, “Now I need help.”

To remain involved, several participants described trying to interpret and adjust to what they saw as the system’s hidden logic, unwritten rules, and expectations. This included staying calm, appearing rational, and avoiding strong emotional expressions so as not to undermine their chances of involvement or risk being sidelined as collaborative partners. One partner described having developed certain phrases he knew would facilitate communication:

I’ve noticed that when I call the staff, I always explicitly acknowledge the rules of confidentiality and the limitations of what they can share with me. Interestingly, this seems to make them more understanding of my needs.

Other participants spoke of withholding or adjusting information to gain access, while others tried to be more assertive to have their concerns taken seriously. Although their strategies differed, many expressed an initial hope for collaboration, grounded in a belief that their knowledge and involvement mattered. At the same time, efforts to stay involved were often accompanied by a recurring sense of insignificance and powerlessness in relation to what they experienced as a large, complex, and at times impersonal system. Several participants described feeling as if they were up against the system, as one mother illustrated when recalling a typical meeting with staff and clinicians:

And then they sat there and talked about their opinions. And we sat there, or at least I did, thinking they have so much knowledge and competence. And it makes you feel incredibly small. But in reality, it’s we who know our daughter best.

Feeling reduced in their capacity to influence their loved one’s care shaped how participants enacted their role as next of kin. This was particularly evident when mistrust or disagreement arose around aspects of care, something several participants experienced. Some described moderating their opinions, including one mother who recalled:

I have always been terrified that the ones in charge of my daughter’s treatment would because they get annoyed with me for asking questions, treating her badly. That’s been a thing, that you become afraid of being critical or asking challenging questions. Because it is they who have the knowledge, in a way. But the situation was really bad, I have to say. I can say that today. Today I dare to say it out loud.

In addition, for several participants, distrust toward healthcare appeared to develop through having either observed or been told about coercive treatment, stigma, or other troubling aspects of care. This further complicated their interactions with the healthcare system. One participant described how both parents and their daughter feared for the daughter’s safety on the ward, and how distrust in the staff’s handling of her self-harm led them to stay as much as they could:

I’m not sure whether they knew how to handle it, but the way she was not cared for left us feeling that we had no other choice than to be there every single day. We left work early and stayed until late in the evening. One of us was always there, while the other was at home with the other children.

Several participants also linked their distrust in the system to uncertainty about treatment progress and a lack of clear treatment rationales, as described by one mother:

The most important thing was that my daughter would get help to recover. For a very long time, in our experience, she didn’t. The self-harm and cutting just escalated, and when that happens, you start to lose faith that the treatment will actually help.

In addition, many participants felt unsure how their loved one’s difficulties were understood within the system, both in diagnostic terms and in relation to who they were as persons. Reflecting on the many diagnoses applied to her daughter’s condition, one mother said:

And then she sort of ended up being given four or five diagnoses. And I don’t have much faith in that. When is a diagnosis supposed to let go? Is it really about piling on diagnoses?

As such, many participants described their attitudes towards healthcare as being marked by a mixture of reliance and mistrust, making the system difficult to navigate, particularly when no other options seemed available. One participant stated: “We’re really grateful to have them [healthcare professionals] because we don’t have anything else.”

Alongside this mixture of reliance and mistrust, some also spoke about feelings of guilt and fear of letting their loved one down, which added further complexity to their experience and their reliance on healthcare. Although healthcare could offer safety or necessary help, several parents described a painful sense that they should have been able to care for their child at home but felt unable to do so. One mother who had left her daughter in supported housing provided by local services recalled:

I just saw an incredibly sad girl. Incredibly sad. She was sort of just standing there in the living room like, “Are you really leaving me?” She didn’t say it, but I felt it, that now I am leaving. I don’t think I even made it past the door before I started crying.

Adding to these tensions, several participants described having limited access to information and limited involvement in decisions, particularly because confidentiality often restricted communication. As a result, the participants frequently relied on what their loved one chose to share after discharge, leaving them uncertain about what had occurred during admission and what responsibility they were expected to carry afterwards. One mother recalled how she experienced her daughter’s discharge from the ward and the responsibility she felt was suddenly placed on her:

When she’s admitted, she gets help there. But when she comes out, it’s us who take over from the moment she walks out the door. And now, I almost feel that when she comes out, she’s worse than before she was admitted, in a way. So, I feel like everything has just become worse and worse.

Her account illustrates how responsibility could shift abruptly back to next of kin, often without clarity about what had happened during treatment or what role they were expected to assume afterwards.

Taken together, these accounts show how next of kin find themselves navigating a powerful system while continuing to feel deeply responsible for their loved one’s wellbeing. Even as healthcare assumed formal responsibility for treatment, participants described a continued need to remain attentive, involved, and ready to act. Therefore, their encounters with the system were marked by a mixture of reliance and uncertainty alongside an ongoing effort to understand their place within this system.

This theme focused on how participants navigate the structures and expectations of the healthcare system; the subsequent themes turn to how these encounters shape their experiences of being recognised and included within the system.

### Theme 2: who do I become when excluded

This theme captures how participants navigate a continuum between moments of recognition and experiences of exclusion frominvolvement and influence in their encounters with healthcare. While some participants recalled instances of inclusion, they more often described feeling sidelined, overlooked, or insufficiently heard. These experiences shaped how participants came to understand themselves in relation to healthcare, influencing their sense of role, value, and place in these encounters.

Experiences of exclusion appeared in many forms and often intersected with participants’ efforts to engage and advocate as described in the previous theme. These experiences ranged from concerns about basic respect and empathy to deeper questions of being valued as a person and as someone who knew their loved one well. Although some accounts related to confidentiality, participants more often described situations in which it was unclear when, how, or to what extent information was shared, both by them and by professionals. Several participants sensed that their commitment was interpreted as overly emotional or overinvolved, whereas others described encounters in which healthcare professionals appeared confident in their own assessments and less open to insights from next of kin. A few participants also felt subtly judged, as if their involvement or way of caring was viewed with suspicion.

Across accounts, participants expressed strong concern about not being heard when trying to convey both their knowledge and the seriousness of their worries. This contrasted with participants’ everyday lives, where they were closely involved in supporting their loved ones and held extensive, experience-based knowledge of their loved one’s situation and needs. Many emphasised that, while healthcare professionals possess specialised expertise, parents and family members know the person best, not only in terms of symptoms or history but as a whole person shaped by relationships, everyday life, and long-term familiarity. Thus, several expressed a desire for this knowledge to be more actively sought and considered, particularly in how their loved one’s difficulties were understood and responded to during treatment and diagnosis. As one father explained:

I think they need to try to spend a bit more time listening to the parents. Especially in the early stages, to get to know the person who’s ill better than they sometimes do. Because whether it’s our case or someone else’s, it’s usually the parents who know their own child best. I’m kind of thinking, sure, a psychologist is skilled and all that. But when a complete stranger comes in, it’s not fixed just like that (snaps fingers), not unless you have a solid background to work from. That’s just my take on it.

Although participants often spoke about not being heard regarding their concerns about how their loved one’s situation would be understood and addressed in care, some also reflected on the deeper personal and existential significance of these experiences. One mother expressed this when describing how it felt to be in the situation: “I felt that my own existence was wiped away. It was like I was retouched out of the picture.” When she elaborated further, she returned to the same feeling of erasure: “At times, I have felt that I ceased to exist. You are there, but you do not really exist.”

Several participants spoke about being physically present but treated as little more than “the mother” or “the one waiting outside,” rather than as someone with insight, resources, or a need to be included. One mother described how sitting in waiting rooms without being acknowledged not only left her uninformed but intensified the doubts and guilt she already carried:

Is it my fault that this is happening? Although people have tried to reassure me that it is not, as a mother, many thoughts inevitably come up. We never talked with anybody at the clinic. It was always only her [my daughter]. We were placed, or I was placed, outside in the waiting area and sat there waiting … So, we didn’t know anything. And not being recognized, it kind of reinforces the feelings of not being a good enough parent. It gets reinforced in a way.

Another recurring aspect of exclusion concerned how participants’ contributions were received in practice. When their involvement was perceived as overly emotional or excessive, their concerns were often dismissed or downplayed rather than taken up and engaged with as meaningful contributions. Participants described how this could affect whether their concerns were taken into account. One sister described how this unfolded when she tried to alert staff that her sister could not be left alone in her room:

I told them I had to leave and that she couldn’t be alone, but no one took me seriously. She ended up sitting there alone for at least an hour … It felt like they saw me as just the emotionally involved sister, and they had to assess what to believe. I understand that it is easy to think we overreact, but it was uncomfortable because I know she can do things when she is alone. Especially now, when she has actually cut herself … I tried to say something, but no one heard me.

One mother recalled an acute situation in which she sensed, with what she described as “every part of her body,” that something was wrong with her daughter after a change in admission status to voluntary admission. She tried to communicate this to staff but felt dismissed. When she became visibly stressed and voiced her worry more firmly, she recalled being told that her behaviour might need to be reported. For these participants, being seen as “too emotional” left them feeling sidelined at moments when the stakes felt highest.

These intense moments also brought to light how meaningful recognition is in next of kin’s experiences. One participant contrasted experiences of non-recognition in encounters related to her child’s self-harm with earlier situations, such as childbirth, where her intuitive, embodied understanding had been acknowledged and trusted. Several described meaningful experiences of being listened to and taken seriously, when they felt valued both as individuals and as contributors in the situation. As one mother reflected:

Just being seen as a human being and not as someone who isn’t worth listening to ... It’s almost like you’re brought back to life again, and you feel that you exist more.

This theme highlighted the importance participants placed on being recognised, or the absence of recognition, not only in terms of potential consequences for their loved one’s wellbeing but also for how they come to understand and value themselves.

Alongside intense moments of feeling unheard or unseen, several participants described more subtle forms of exclusion. Across these experiences, participants emphasised the significance of being acknowledged as someone who matters in their loved one’s care.

### Theme 3: the ones who stand by

This theme concerns how next of kin described their own struggles and needs becoming less visible in the shadow of the crisis. While attention was directed toward the person receiving treatment, those standing beside them often felt that their own needs were overlooked. As one mother put it: “In ten years, only one person has asked how I’m doing.” Another mother expressed this more fully:

As parents, we were a bit forgotten. And I think the siblings should have received more attention than they did. They were offered something when the crisis first started, but this has been going on for years. And I think it’s hard for them [siblings] to say, “Now I need someone to talk to. Can I get some help?” If someone had just said, “We have this group for adolescents.” But there wasn’t much information.

Interviewer: So, there wasn’t much communicated to you about those kinds of support options?

Mother: No. I would say that, for us as parents, there was nothing.

Although not everyone used equally strong words, a similar sense of invisibility ran through many of the interviews. Several participants described how their worries, exhaustion, and vulnerability were rarely addressed. Attending to their own needs was often experienced as secondary and only considered when time and energy allowed, which they felt they did not have. As one father explained:

Part of the challenge of being a next of kin is that you don’t focus on yourself or your own needs. Your attention is entirely on the child, the partner, or whoever is affected. That’s where your energy goes.

Support was frequently described as something one had to actively pursue, but simply holding everyday life together already required all their available strength. As one mother put it: “Support should have been offered to us.”

Others spoke of uncertainty about where to turn or who to contact. A sibling explained:

You kind of get this, like, ‘if you need someone to talk to, just get in touch,’ sort of thing. And then you’re left thinking, okay, but who am I supposed to contact? How does this even work?

Together, these accounts show how responsibility for seeking help was often placed on next of kin themselves, even in the midst of exhaustion. The expectation to self-initiate support reinforced a sense that their struggles fell outside the main frame of care.

This sense of standing outside was also visible in how participants described the wider family. Several parents expressed ongoing concern for siblings and how the situation affected them. Attention was understandably directed toward the child in crisis, but other children in the family often received little follow-up as the years passed. Sibling participants reflected on how family life changed around them. Some described receiving less attention and care during critical periods, and some noted that, particularly at a younger age, it had been difficult to recognise or articulate their own needs. As one sibling explained:

I was so young and had no idea what I needed or what should have been addressed or discussed. That seems to be a recurring pattern in healthcare and is something that I find quite stupid when it comes to how the healthcare system works.

Her reflection points to how the absence of proactive outreach could leave siblings navigating complex emotions without language or guidance. Some described how the effects of this imbalance lingered into adulthood.

Similarly, parents who were or had been married described the toll the situation had taken on their relationship. The cumulative strain gradually reduced space for communication, intimacy, and a sense of ordinary family life, leading in some cases to marital breakdown or divorcee. Several participants emphasised the need for more family-oriented and proactive forms of support that would recognise not only the individual in crisis but those living alongside them.

Many participants described their own needs as being set aside in the crisis, whereas some participants recalled moments when professionals acknowledged them as persons with concerns and limits of their own. One mother described feeling genuinely recognised when a clinician addressed her directly, acknowledged her efforts and needs, and invited her perspective. She reflected on how this moment affected her:

And there’s such relief in realizing, ‘oh, I don’t have to carry all of this alone,’ because it’s almost like you can’t breathe from the weight of it. You have to balance and contain everything at once. No one can understand it before they’re in such a situation.

Several participants also spoke about the value of meeting others in similar situations. Although none had experience with groups for next of kin of individuals who self-harm, many imagined that such spaces might offer recognition and relief from isolation. They described a hope that others who carried comparable burdens might provide mutual understanding, validation of difficult emotions, and perhaps a sense of hope through shared experience. A few had participated in next-of-kin groups in other contexts and described them as meaningful. One participant described how important this had been for her ability to cope:

It has been very important for me to be able to share my experiences with someone who was in a similar situation. If I hadn’t had anyone to vent to, I think I would have gone completely crazy. Of course, there are friends and other family members, but it’s not easy to share this kind of experience because it’s so hard for others to understand if they haven’t been in a similar situation.

At the same time, one participant noted that such groups could sometimes take on an activist or resentful tone towards the system, which was not always helpful. Across accounts, the value seemed to lie less in advocacy and more in the possibility of mutual recognition and shared understanding.

This theme highlighted the limited recognition of the needs and vulnerabilities of those who stand by as attention is primarily directed toward the person in distress. Moments of acknowledgement, when the next of kin were recognised as persons in their own right with legitimate needs, stood out as rare but meaningful. These accounts also pointed to the importance of more proactive and relational forms of support, through professional outreach or connection with others in similar circumstances.

## Discussion

The aim of this study was to explore how next of kin to individuals struggling with severe and prolonged self-harm experience and make sense of their encounters with healthcare services. Our results suggests that these encounters are not experienced merely as coordination challenges but as relational and moral events in which next of kin’s credibility, role, and personhood are continuously negotiated. By reading participants’ accounts through the lenses of epistemic injustice (Fricker, 2007) and mattering (Prilleltensky, 2014), we offer conceptual language for why these interactions can feel emotionally and existentially demanding and how they can shape both the next of kin’s involvement in care and their wellbeing.

Consistent with prior research, participants described persistent fear for their loved one, high emotional strain, and an ongoing responsibility that did not diminish when formal services were involved (Abou Seif et al., 2022; Martin et al., 2024). They also described navigating complex systems, unclear roles, and unmet needs for information and support, especially during transitions between inpatient and community-based care, echoing previous reports in which relatives often felt excluded from communication and decision-making (Dietrich et al., 2025; Stewart et al., 2018; Zhao et al., 2023). This body of work has been crucial in making visible the burdens placed on next of kin and highlighting structural and relational challenges within mental healthcare. The present study adds to this literature by foregrounding what these barriers come to mean for next of kin: how exclusion and non-recognition affect their sense of legitimacy as contributors, their trust in services, and their sense of self.

A cross-cutting tension in participants’ stories was the simultaneous dependence on professional care and the felt need to remain vigilant and involved. Entrusting care to the system required a degree of surrender that many participants experienced as risky, particularly when they perceived coercion, stigma, or inadequate attention to escalating self-harm risk. To remain involved, some learned to “speak the system’s language” and regulate emotional expression; others experienced that strong feelings were interpreted as overinvolvement and reduced their influence. Such experiences illuminate how the conditions of interaction, including time pressure, uncertainty, professional authority, and institutional routines, shape which perspectives are heard, regarded as credible, and ultimately influence care.

### Epistemic injustice and experiential knowledge

Through ongoing involvement in everyday life, next of kin may develop detailed knowledge of their loved one's history, everyday functioning, signs of escalating distress, and what tends to help or worsen distress (Hughes et al., 2017; Lindgren et al., 2010; Stewart et al., 2018; Townsend et al., 2023). Participants in the present study often described this knowledge as emerging through close involvement and, at times, as being “felt in the body” before it could be articulated. Such accounts resonate with phenomenological perspectives emphasising how understanding arises through lived and embodied engagement with others (Finlay, 2005). Yet participants often experienced that this form of knowing was not invited into clinical conversations, or that it was treated as less credible than professional judgments.

Fricker (2007) concept of epistemic injustice helps clarify why this was experienced as more than ordinary disagreement. In healthcare, epistemic injustice has been used to describe how patients are wronged as knowers through credibility deficits and restricted opportunities for sense-making (Carel & Kidd, 2014; Crichton et al., 2017; Côté, 2024). Our results suggest that similar dynamics can affect next of kin; they may be disadvantaged in their capacity to contribute knowledge about the person in care, even when that knowledge is highly relevant for understanding risk and everyday functioning. Testimonial injustice is relevant when credibility is unjustly deflated because of prejudicial assumptions about the speaker (Fricker, 2007). Several participants felt that their contributions were discounted by the professionals because they were “too emotional,” “too involved,” or treated as biased by definition.

Such accounts resonate with work suggesting that credibility may be undermined through pathocentric attitudes, often manifesting as diagnostic stigma, particularly in contexts in which self-harm is associated with diagnoses that carry negative stereotypes (Carel & Kidd, 2014; Crichton et al., 2017; Kidd et al., 2025; Sheehan et al., 2016). Although next of kin are not patients, research on stigma by association and courtesy stigma suggests that close family members may be affected by spillover processes that position them as suspect, responsible, or emotionally unreliable (Koro-Ljungberg & Bussing, 2009; Van Der Sanden et al., 2015). In this light, the recurring description of being treated as “just the mother” or “the emotionally involved sister” can be read as more than interpersonal mismatch; it points to patterned credibility dynamics operating within a broader stigma ecology.

Hermeneutic injustice concerns constrained opportunities for shared sense-making, including limited interpretive resources or limited space to make experiences intelligible within institutional settings (Fricker, 2007; Kidd & Carel, 2018). In the present study, hermeneutic injustice was visible in how meetings were time pressured, structured around professional categories, and bounded by confidentiality, leaving little room to translate relational knowledge into the treatment process. Participants described struggling to convey subtle changes in the posture, tone, or routines of their loved one, or a sense that something was wrong, because such observations did not easily fit institutional language or templates. These difficulties suggest that knowledge grounded in everyday relational and embodied experience may not easily fit within healthcare settings that prioritise biomedical forms of explanation (Kidd & Carel, 2018; Kidd et al., 2025). This aligns with arguments that healthcare institutions can be epistemically opaque to outsiders, limiting families’ ability to understand, evaluate, and contribute to decision-making (Carel & Kidd, 2023). Some accounts also resonate with contributory injustice, where individuals are not only misunderstood but are denied uptake of the interpretive resources they already possess because dominant frameworks do not recognise these as legitimate contributions (Tate, 2019). However, not all forms of exclusion necessarily reflect injustice (Nielsen et al., 2025). In healthcare settings, confidentiality and clinical risk considerations may legitimately limit information sharing (Carel & Kidd, 2014). However, the participants’ accounts suggest that experiences of not being heard were often not primarily about the absence of information but about the absence of fair and just engagement: unclear expectations, minimal acknowledgement, and limited opportunities to contribute even when contributing was possible. This aligns with research showing that institutional routines can systematically narrow whose accounts are treated as relevant (Côté, 2024), with emotionally expressed narratives sometimes received as catharsis rather than epistemic insight (Kok et al., 2022). Taken together, the epistemic injustice lens helps explain why participants described both frustration and destabilization: Their capacity to act responsibly was undermined in contexts in which they considered themselves to be carrying significant responsibility.

### Mattering

Epistemic injustice clarified how credibility and sense-making can be constrained, whereas the concept of mattering highlights what is existentially at stake in these encounters. Mattering involves both feeling valued and having opportunities to add value (Prilleltensky, 2020; Prilleltensky et al., 2023). This duality maps closely onto our themes. Participants sought involvement in care processes not only to obtain information but to contribute their intimate knowledge and act in their loved one’s best interests. When they experienced being excluded, dismissed, or treated as peripheral, next of kin lost not only opportunities for involvement and influence, but also opportunities to contribute and matter in ways that affirmed their role and moral commitment. At the same time, many participants described a parallel need to matter as persons in their own right, to be seen as human beings affected by prolonged strain, not merely as extensions of the patient.

Placing mattering alongside epistemic injustice foregrounds fairness as a core issue, not an optional “family-friendly” add-on. In our results, fairness appears in several forms: epistemic justice, concerning who is treated as a credible source of knowledge (Fricker, 2007); procedural justice, concerning who is included in processes that affect them (Lind, 2019; Tate, 2019; Von Heimburg & Ness, 2021); and relational fairness, concerning whether people are met with dignity and moral regard (Fricker, 2007; Honneth, 2004; Lind, 2019; Prilleltensky et al., 2025). These forms of justice are closely linked to wellbeing (Prilleltensky et al., 2023, 2025). When the next of kin’s sense of mattering was supported through attentive listening, clear communication, and genuine inclusion, participants described relief, renewed capacity, and a sense of being “brought back to life.” When mattering was undermined, some described experiences closer to anti-mattering (Flett et al., 2022): feeling erased, insignificant, or treated as though their involvement carried less legitimacy. Such experiences may be particularly consequential because they occur in a context where guilt, self-doubt, and emotional exhaustion are already common burdens for family members supporting a loved one who self-harms (Curtis et al., 2018; Martin et al., 2024; Mughal et al., 2022). This interpretation resonates with research linking anti-mattering to negative self-appraisals and psychological distress (Flett et al., 2022; Krygsman et al., 2022).

Our results also point to a wider wellbeing ecology: Next of kin’s ability to remain well is not separate from the patient’s wellbeing or the system’s functioning. Families were already doing substantial safeguarding and emotional labour, especially at discharge and between healthcare contacts. When systems rely on next of kin for continuity and safety while simultaneously limiting recognition and support, a fairness problem arises; responsibility is shifted without corresponding voice, resources, or acknowledgement. In such conditions, involvement risks becoming extractive rather than collaborative. A mattering lens helps articulate why this is not only inefficient but ethically troubling, as it undermines both dimensions of mattering by reducing opportunities to add value and eroding the sense of being valued (Prilleltensky, 2020).

Peer support emerged as an important counterpoint. Participants described (or imagined) peer contexts as spaces where their experiences were intelligible and credible without extensive explanation and where they could both receive and offer value. From a mattering perspective, these contexts can restore both dimensions of mattering: being valued through recognition and adding value through mutual support (Prilleltensky, 2020; Prilleltensky et al., 2023). From a justice perspective, peer spaces also redistribute interpretive power by offering shared language for the experience and reducing the need to translate distress into institutionally sanctioned forms. The expressed need for such spaces suggests that family-oriented care should include not only informational interventions but relational and community-based support that can sustain belonging, recognition, and meaning.

### Strengths, limitations, and future directions

The strength of this study lies in its in-depth, experience-near accounts from next of kin who had supported a loved one through severe and prolonged self-harm over many years, allowing us to explore how recognition, exclusion, and responsibility unfold over time. Nevertheless, our interpretive analysis cannot determine clinicians’ intentions or the full institutional constraints that shaped each encounter. Some situations that participants experienced as restricting their opportunities for involvement and contribution may have been driven by legitimate confidentiality requirements or by situational risk management. Therefore, our claim is not that all exclusion constitutes epistemic injustice, but that next of kin’s accounts reveal recurring credibility and sense-making dynamics that are plausibly understood as epistemic and relational unfairness, and that these dynamics have consequences for wellbeing.

As recruitment required consent from the person who self-harmed, next of kin in the most conflicted relationships may be underrepresented. In addition, the study is situated in a Norwegian context, and legal and organisational frameworks for the involvement of next of kin differ across countries. Future research could compare how different service models and legal regimes shape mattering and fairness for families. Intervention studies could test whether structured involvement practices, proactive family support, and peer-based support foster experiences of recognition, mattering, and meaningful inclusion among next of kin, and whether these experiences contribute to improving wellbeing outcomes for both next of kin and the person receiving care. This reciprocal perspective is also reflected in recent qualitative studies of relatives' experiences of voluntary brief admission for individuals who self-harm, which suggest that interventions directed at patients may also have important implications for next of kin by reducing family burden, increasing predictability, and supporting relational wellbeing (Hultsjö et al., 2023; Lindkvist et al., 2024; Värnå et al., 2025).

## Conclusion

Taken together, the results suggest that improvements to next-of-kin experiences require more than the provision of information. Health care services need practices that actively cultivate epistemic and relational justice. First, early and explicit clarification of roles, boundaries, and what confidentiality does and does not prevent may reduce institutional opacity and help next of kin understand how to contribute to the care of their loved one without fearing retaliation or missteps. Second, health care services can create structured opportunities for next of kin to contribute observations about risk, change, and what helps, particularly at admission, during crises, and at discharge. Such opportunities can be designed so that the next of kin’s experiential knowledge is treated as a resource that complements clinical assessment rather than as knowledge that remains peripheral to care processes. Third, mental healthcare staff can adopt routines that recognise next of kin as persons who are affected. Brief check-ins, proactive offers of support, and clear pathways to family or caregiver services may reduce the burden of having to self-initiate help when the next of kin’s capacity is depleted.

At the level of clinical culture, our findings invite reflection on how emotional expression is interpreted. Rather than treating emotion as a credibility deficit, clinicians can recognise distress as contextually rational and potentially informative, especially in situations of sustained threat and uncertainty. Doing so may reduce the self-reinforcing cycle in which not being heard intensifies emotion, which then further reduces credibility (Carel & Kidd, 2023). Finally, health care services should consider integrating or partnering with peer support initiatives for next of kin given their potential to support mattering, fairness, and wellbeing through recognition and mutual aid.

## Supplementary Material

Supplementary Material COREQ report.docx

## Data Availability

The participants of this study did not give written consent for their data to be shared publicly, so due to the sensitive nature of the research supporting data is not available.
