# Supplementary material for: Next of kin's experiences of healthcare encounters in the context of severe self-harm: “We know them best”
Source: Int J Qual Stud Health Well-being. 2026 Aug 12;21(1):2712680. doi: 10.1080/17482631.2026.2712680 (PMC13470909; doi:10.1080/17482631.2026.2712680)
Supplement: Supplementary file 1 [file ZQHW_A_2712680_SM9968.docx]

Supplementary File 1: COREQ checklist

Consolidated criteria for reporting qualitative studies (COREQ): 32-item checklist

Developed from:

Tong A, Sainsbury P, Craig J. Consolidated criteria for reporting qualitative research (COREQ): a 32-item checklist for interviews and focus groups. International Journal for Quality in Health Care. 2007. Volume 19, Number 6: pp. 349 – 357

| **Item No** | | **Guide Questions/Description** | **Reported on Page #** |  |  |
| --- | --- | --- | --- | --- | --- |
| **Domain 1: Research team and reflexivity** | | | |  |  |
| **Personal Characteristics** | | | |  |  |
| 1. Interviewer/ facilitator | | Which author/s conducted the interview or focus group? | P. 8: *In-depth interviews were conducted by (names removed for blind review) the first author (name removed for blind review), with two co-authors (names removed for blind review) attending one interview each to facilitate a shared familiarity with the material within the research team.* |  |  |
| 2. Credentials | | What were the researcher’s credentials? E.g., PhD, MD | *P. 10: The research team includes..*(Description of research team removed for blind review) |  |  |
| 3. Occupation | | What was their occupation at the time of the study? | P. 1, researcher affiliation is reported |  |  |
| 4. Gender | | Was the researcher male or female? | *P. 10: P. 10: The research team includes..*  *(Description of research team removed for blind review)* |  |  |
| 5. Experience and training | | What experience or training did the researcher have? | *P. 10: The research team..* *(Description of research team removed for blind review)* |  |  |
| **Relationship with participants** | | | |  |  |
| 6. Relationship established | | Was a relationship established prior to study commencement? | Although one participant was previously known to the interviewer, no established relationship existed prior to study commencement. This is not reported in the manuscript to protect confidentiality. |  |  |
| 7. Participant knowledge of the interviewer | | What did the participants know about the researcher? e.g. personal goals, reasons for doing the research? | P. 11: *They were informed about the study's purpose, the first author's role as a PhD candidate and employment at the local hospital,* |  |  |
| 8. Interviewer characteristics | | What characteristics were reported about the interviewer/facilitator? e.g. Bias, assumptions, reasons and interests in the research topic | *P. 10: In addition to our professional backgrounds, we reflected on how personal experiences, including being next of kin, shaped our approached the material. For example, before engaging in the formal analysis, the research team held a dedicated discussion to identify and reflect on the professional and personal experiences we brought to the study and how these might shape our attentiveness to particular meanings in the data.* |  |  |
|  | **Domain 2: study design** | | |  |  |
|  | **Theoretical framework** | | |  |  |
|  | 9. Methodological orientation and Theory | What methodological orientation was stated to underpin the study? e.g. grounded theory, discourse analysis, ethnography, phenomenology, content analysis | P. 6: *This qualitative study was situated within an interpretivist paradigm (Cuthbertson et al., 2020) and was informed by a phenomenological orientation attentive to participants' lived experiences and meaning-making (Dahlberg et al., 2007; Van Manen, 2014).* |  |  |
|  | **Participant selection** | | |  |  |
|  | 10. Sampling | How were participants selected? e.g., purposive, convenience, consecutive, snowball | P. 7: *Only next of kin of adults (18 years or older) who self-harmed were eligible for participation. In most cases, healthcare professionals informed individuals struggling with self-harm about the study. Next of kin were approached only after the person who self-harmed and their next of kin had agreed that the next of kin could be approached about the study.* |  |  |
|  | 11. Method of approach | How were participants approached? e.g., face-to-face, telephone, mail, email | P. 7: *In some cases, the person who self-harmed informed their next of kin about the study, and interested next of kin contacted the first author directly. In other cases, interested next of kin were contacted by telephone by the first author after agreeing to be contacted.* |  |  |
|  | 12. Sample size | How many participants were in the study? | P. 6: *The participants were 15 next of kin, all of whom were family-members, closely involved with a child, partner, or sibling who had struggled with severe self-harm for between 5 and 15 years*. |  |  |
|  | 13. Non-participation Setting | How many people refused to participate or dropped out? Reasons? | NR |  |  |
|  | 14. Setting of data collection | Where was the data collected? e.g., home, clinic, workplace | P. 8: *Most interviews were conducted in hospital offices in accordance with participant preference. One interview was conducted in an office facility provided by the participant. Two participants were interviewed via video due to geographic distance. Video interviews were conducted using a secure digital platform provided by the hospital where the study was conducted.* |  |  |
|  | 15. Presence of nonparticipants | Was anyone else present besides the participants and researchers? | P. 8: *In-depth interviews were conducted by the first author (name removed for blind review), with two co-authors (names removed for blind review) attending one interview each to facilitate a shared familiarity with the material within the research team* |  |  |
|  | 16. Description of sample | What are the important characteristics of the sample? e.g. demographic data, date | Pp. 6-7 (table) |  |  |
|  | **Data collection** | | |  | No |
|  | 17. Interview guide | Were questions, prompts, and guides provided by the authors? Was it pilot tested? | P. 8. A semi-structured interview guide was developed by the authors in collaboration with user representatives. It was not formally pilot tested. |  |  |
|  | 18. Repeat interviews | Were repeat interviews carried out? If yes, how many? | P. 8: *Twelve participants were interviewed twice, while follow-up interviews were not conducted with three participants due to practical circumstances.* |  |  |
|  | 19. Audio/visual recording | Did the research use audio or visual recording to collect the data? | P. 9: *All interviews were audio-recorded and transcribed verbatim by the first author with assistance from open-source speech recognition tool Whisper (Whisper, n.d.) used within the University of Oslo’s secure TSD platform (Services for Sensitive Data). Anonymized transcripts were uploaded to NVivo (version 14), for data management.* |  |  |
|  | 20. Field notes | Were field notes made during and/or after the interview or focus group? | NR. Reflective memos were written during data collection, but this was not undertaken systematically and is not reported in the manuscript. |  |  |
|  | 21. Duration | What was the duration of the interviews or focus group? | P. 8: *Initial interviews typically lasted around 90 minutes. Follow-up interviews varied in length but were generally shorter than the initial interviews.* |  |  |
|  | 22. Data saturation | Was data saturation discussed? | P. 7-8. The study draws on the concept of information power (Malterud et al., 2016) to justify sample size rather than the concept of data saturation. |  |  |
|  | 23. Transcripts returned | Were transcripts returned to participants for comment and/or correction? | No |  |  |
|  | **Domain 3: analysis and findings** | | |  |  |
|  | **Data analysis** | | |  |  |
|  | 24. Number of data coders | How many data coders coded the data? | P. 9: *Initial coding by the first author was predominantly semantic in order to stay close to participants’ accounts.* |  |  |
|  | 25. Description of the coding tree | Did the authors provide a description of the coding tree? | No. The analytic process is described in the manuscript (Pp. 9-10), but a coding tree is not presented |  |  |
|  | 26. Derivation of themes | Were themes identified in advance or derived from the data? | Pp. 9 -10. Themes were developed inductively through an iterative reflexive thematic analysis, informed by close engagement with participants' accounts |  |  |
|  | 27. Software | What software, if applicable, was used to manage the data? | P. 9: *All interviews were audio-recorded and transcribed verbatim by the first author with assistance from open-source speech recognition tool Whisper (Whisper, n.d.) used within the University of Oslo’s secure TSD platform (Services for Sensitive Data). Anonymized transcripts were uploaded to NVivo (version 14), for data management.* |  |  |
|  | 28. Participant checking | Did participants provide feedback on the findings? | No (p. 10-11). User representatives contributed to discussions of developing interpretations, but study participants did not provide feedback on the findings. |  |  |
|  | **Reporting** | | |  |  |
|  | 29. Quotations presented | Were participant quotations presented to illustrate the themes/findings? Was each quotation identified? e.g., participant number | Pp. 13-23. Yes quotations were presented to illustrate findings. Quotations were not individually identified to protect participant anonymity |  |  |
|  | 30. Data and findings consistent | Was there consistency between the data presented and the findings? | Yes (pp. 12–24). Participant quotations are presented throughout the Results section to illustrate and support the findings. |  |  |
|  | 31. Clarity of major themes | Were major themes clearly presented in the findings? | Yes. Pp. 12-24 |  |  |
|  | 32. Clarity of minor themes | Is there a description of diverse cases or a discussion of minor themes? | Yes (pp. 12–24). Variation and less common experiences are described within themes. |  |  |
